# Frequent Fires in Ancient Shrub Tundra: Implications of Paleorecords for Arctic Environmental Change

**DOI:** 10.1371/journal.pone.0001744

**Published:** 2008-03-05

**Authors:** Philip E. Higuera, Linda B. Brubaker, Patricia M. Anderson, Thomas A. Brown, Alison T. Kennedy, Feng Sheng Hu

**Affiliations:** 1 College of Forest Resources, University of Washington, Seattle, Washington, United States of America; 2 Department of Earth and Space Sciences and Quaternary Research Center, University of Washington, Seattle, Washington, United States of America; 3 Lawrence Livermore National Laboratory, Center for Accelerator Mass Spectrometry, Livermore, California, United States of America; 4 Department of Earth Sciences, Montana State University, Bozeman, Montana, United States of America; 5 Department of Plant Biology, University of Illinois, Urbana, Illinois, United States of America; 6 Department of Geology, University of Illinois, Urbana, Illinois, United States of America; Centre National de la Recherche Scientifique, France

## Abstract

Understanding feedbacks between terrestrial and atmospheric systems is vital for predicting the consequences of global change, particularly in the rapidly changing Arctic. Fire is a key process in this context, but the consequences of altered fire regimes in tundra ecosystems are rarely considered, largely because tundra fires occur infrequently on the modern landscape. We present paleoecological data that indicate frequent tundra fires in northcentral Alaska between 14,000 and 10,000 years ago. Charcoal and pollen from lake sediments reveal that ancient birch-dominated shrub tundra burned as often as modern boreal forests in the region, every 144 years on average (+/− 90 s.d.; n = 44). Although paleoclimate interpretations and data from modern tundra fires suggest that increased burning was aided by low effective moisture, vegetation cover clearly played a critical role in facilitating the paleofires by creating an abundance of fine fuels. These records suggest that greater fire activity will likely accompany temperature-related increases in shrub-dominated tundra predicted for the 21^st^ century and beyond. Increased tundra burning will have broad impacts on physical and biological systems as well as on land-atmosphere interactions in the Arctic, including the potential to release stored organic carbon to the atmosphere.

## Introduction

**Figure 1:**
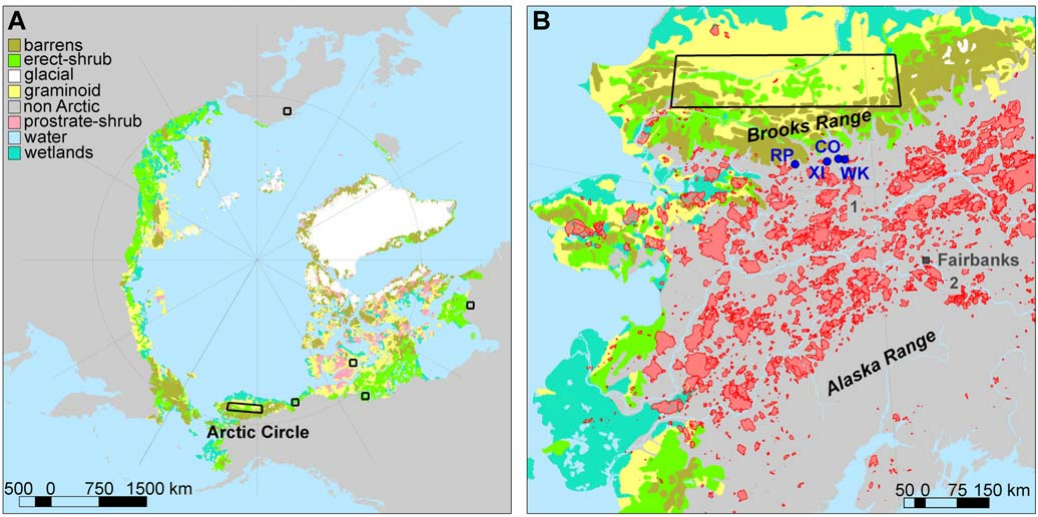
Distribution of modern circumpolar Arctic tundra [15], Alaskan fires from CE 1950–2005, and sites referred to in the text. (A) Black rectangles indicate circumpolar regions showing recent increases in shrub densities and/or extent [10]. (B) Alaskan fires from CE 1950–2005 (red polygons) in tundra and boreal forest. Fires burned only 3% percent of Alaskan tundra, representing 6% of the total area burned in the state. Blue dots identify lakes used in this study: Ruppert (RP) and Xindi (XI) lakes contain records of fire and vegetation from the Shrub Tundra Zone; Ruppert, Code (CO), and Wild Tussock (WK) lakes contain records from the Boreal Forest Zone (5.5-0 ka BP). Sediment-charcoal records from Sithylemenkat Lake (1) [19] and Lost Lake (2) [18] also show qualitative evidence of increased fire activity within the Shrub Tundra Zone.

Tundra and boreal ecosystems store one third of the world's soil carbon [1]. The fate of this vast carbon stock has become a major concern to global-change scientists because its release to the atmosphere could exacerbate CO_2_–related climate change [2]–[6]. Unfortunately, uncertainty about a number of ecosystem processes hampers predictions of future tundra carbon cycling and the potential consequences to the climate system. One of the most important processes is how vegetation and climate change will alter fire regimes of tundra regions [2], [6], [7]. Available evidence suggests that ongoing vegetation and climate change could significantly increase the rate of burning in northern tundra [8], which is currently dominated by low-biomass communities (graminoids, herbs, and dwarf shrubs) that seldom burn [e.g. only 3% of Alaskan tundra burned between CE 1950 and 2005; Fig. 1; 9]. In particular, a marked increase in shrub abundance and density, likely resulting from climate warming [10], is changing the physiognomic structure of arctic and subarctic regions. Shrubby growth forms increase the abundance of fine fuels available for burning, and in light of 3–5°C warming predicted over the next century [8] such fuel changes could result in fire regimes vastly different from those in modern tundra. Unfortunately, short observational fire records [e.g. 48 and 57 years in Canada and Alaska; 9,11], a lack of fire-history studies, and the possibility of novel future vegetation [12] result in little information to evaluate how tundra fire regimes may respond to future climate and vegetation change. The paleoecological approach circumvents these limitations and offers the only way to obtain long-term empirical records of fire and vegetation change relevant for understanding tundra fire regimes under future climate and vegetation scenarios.

Here we present fire and vegetation reconstructions from northcentral Alaska that document frequent fires in shrub tundra during the late-glacial and early-Holocene periods (14-10 ka BP [ka BP = thousand calendar years before present, CE 1950]). Vegetation and climate controls of these unusual fire regimes are inferred from paleovegetation records from each of two sites and from regional paleoclimate interpretations for this period. We also present an analysis of the climate space occupied by modern tundra vegetation and modern tundra fires in Alaska (CE 1950–2004). This analysis provides additional support for the climate-fire relationships inferred from the paleo-data.

## Results

**Figure 2:**
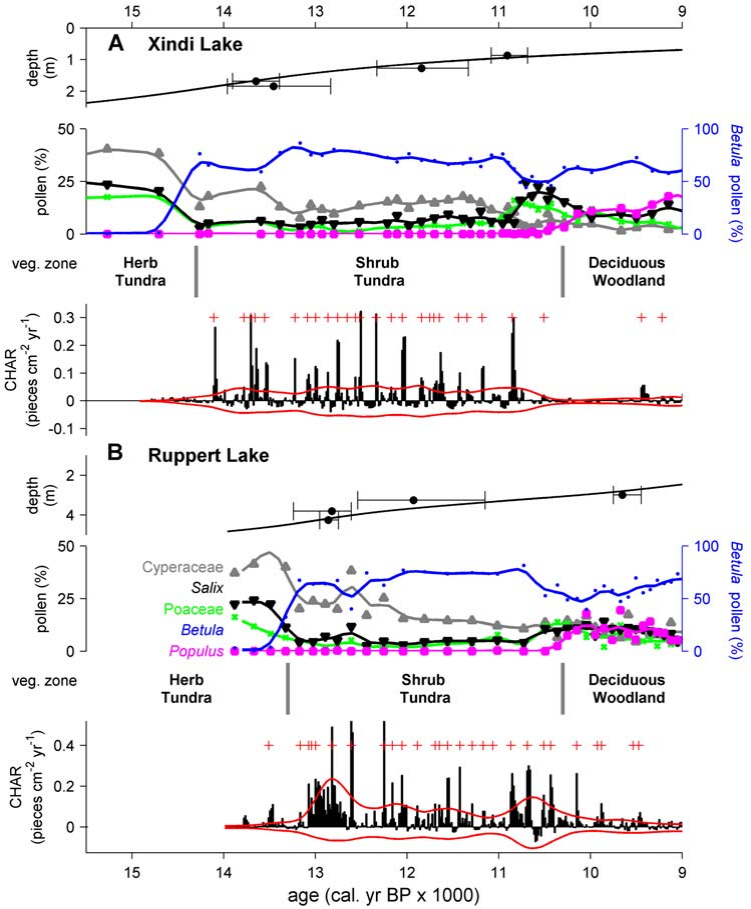
Fire and vegetation reconstructions from northcentral Alaska. Chronology, pollen stratigraphy, inferred vegetation, and high-frequency variations in charcoal accumulation rates (CHARs) from (A) Xindi Lake and (B) Ruppert Lake. Pollen percentage curves are smoothed to 500 years and color coded. CHAR records represent residuals after removing 500-year trends, and red lines around CHAR = 0 are thresholds identifying noise-related variations. Red plus marks identify CHAR peaks exceeding the positive threshold (and a minimum-count screening; see Materials and Methods) and are interpreted as local fire events. At both sites CHARs and CHAR peaks increase distinctly with the rise in *Betula* pollen percentages, marking the transition from the Herb Tundra Zone to the Shrub Tundra Zone.

Trends in charcoal accumulation rates (pieces cm^−2^ yr^−1^, CHARs) correspond markedly with shifts in pollen assemblages at Xindi and Ruppert lakes (Fig. 2). Both records start in herb-dominated tundra (Herb Tundra Zone), indicated by high pollen percentages of Cyperaceae (sedge), Poaceae (grass), and minor herb taxa (e.g. *Artemisia* [wormwood], data not shown). Raw CHARs are low (medians = 0.01 and 0.00 pieces cm^−2^ yr^−1^) with few identified peaks in the detrended series (Fig. 2), suggesting little or no burning in the late-glacial herb tundra near these sites. Increases in CHARs (medians = 0.05 and 0.02 pieces cm^−2^ yr^−1^) and the frequency of peaks in the detrended series coincide with a prominent rise in *Betula* (birch) pollen percentages (from <5 to 50–75%; 14.3 and 13.3 ka BP at Xindi and Ruppert lakes, respectively), which marks the expansion of *Betula* shrubs in the study area (Fig. 2). These pollen assemblages (Shrub Tundra Zone) have higher *Betula* percentages than pollen assemblages from modern tundra in North America [13] (e.g. 70% vs. 40%) and are thought to represent extensive thickets of tall (>1 m) *Betula glandulosa* [resin birch, inferred from measurements of pollen morphology, 14]. The inferred vegetation of the Shrub Tundra Zone contrasts with the majority of modern circumpolar Arctic tundra, where only 12% of the area contains shrubs taller than 0.4 m [i.e. Low-shrub tundra; 15]. However, the vegetation structure of the Shrub Tundra Zone may be analogous to future Arctic tundra, which is predicted to have a major component of >0.5-m tall *Betula*, *Salix* (willow), and *Alnus* (alder) shrubs [10], [16]. Deciduous woodlands (Deciduous Woodland Zone), identified by samples with >10–20% *Populus* (poplar) pollen, characterized the vegetation from 10.5-9.0 ka BP (Fig. 2). As in the Herb Tundra Zone, the low raw CHARs (medians = 0.02 and 0.01 pieces cm^−2^ yr^−1^) and few peaks in the detrended series suggest less frequent fires as compared to the Shrub Tundra Zone.

**Figure 3:**
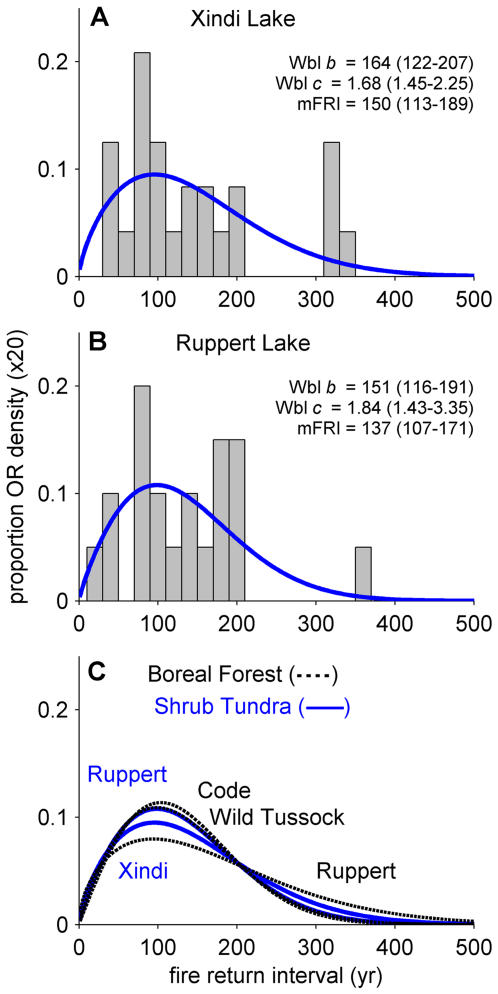
Fire return intervals (FRIs) from the Shrub Tundra Zone and the conifer-dominated Boreal Forest Zone (5.5-0 ka BP). FRIs from the Shrub Tundra Zone at (A) Xindi Lake and (B) Ruppert Lake with fitted Weibull models (blue lines). Weibull (Wbl) *b* (yr) and *c* (unitless) parameters, and the mean FRI (mFRI; yr) all include 95% confidence intervals. (C) Weibull models from the Shrub Tundra Zone (blue solid lines) and the Boreal Forest Zone (black dashed lines). All FRI distributions presented are statistically similar based on likelihood-ratio tests (p>0.29; see Results). The Weibull *b* and *c* parameters, and mFRI for Ruppert (boreal forest), Code, and Wild Tussock lakes are 188 (147–239), 150 (123–178), and 149 (123–174); 1.53 (1.31–2.06), 1.85 (1.52–2.60), and 1.96 (1.61–2.75); 171 (135–216), 135 (113–160), and 135 (113–157), respectively.

Estimated fire frequencies within the Shrub Tundra Zone (Figs. 2, 3) were much higher than in modern tundra [9], [11] (Fig. 1). Fire events (i.e. CHAR peaks) occurred on average (95% CI) every 150 (113–189) years at Xindi Lake and 137 (107–171) years at Ruppert Lake, with high variability around these means (fire return intervals [FRIs] range from 30–360 yr; Fig. 3). FRI distributions at these two sites were statistically indistinguishable during this period (p = 0.60, n = 24, 20) and from FRI distributions in the late-Holocene boreal forests around Ruppert, Code, and Wild Tussock lakes (p ranges from 0.29–0.99, n ranges from 20–39; see Materials and Methods; Fig. 3; Fig. S1). The fire-vegetation relationships observed at Ruppert and Xindi lakes during the Shrub Tundra Zone are likely regional in scale, as this tundra type is documented in a large network of pollen and macrofossil records in northcentral Alaska [12], [13], [17], and high fire activity has been qualitatively inferred from discontinuous charcoal records at other sites in interior Alaska [18], [19] (Fig. 1B).

## Discussion

**Figure 4:**
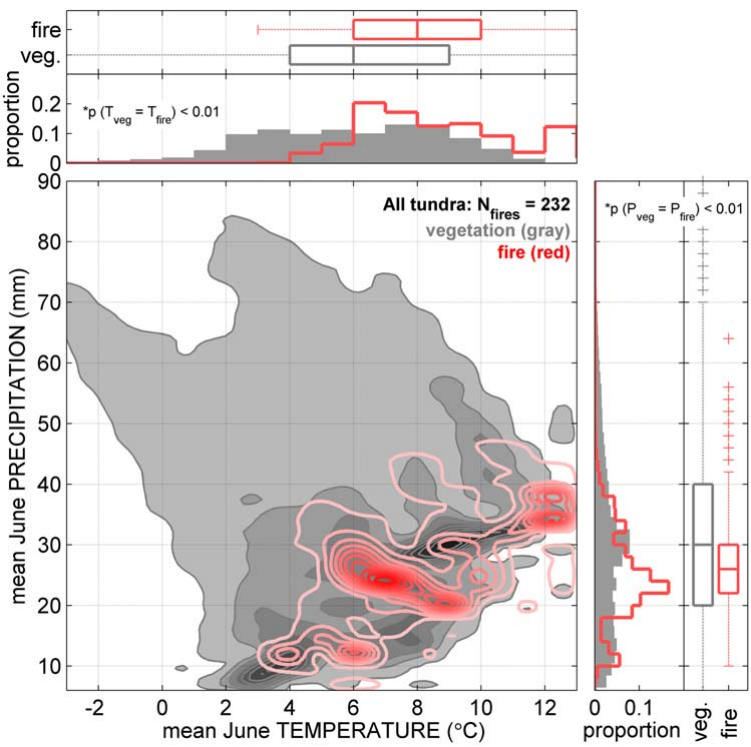
Climate space occupied by Alaskan tundra in the circumpolar Arctic vegetation map [15] and area burned within the same region from CE 1950–2005. Darker shades represent a greater proportion of total tundra vegetation (gray) or total area burned (red) within the climate space. Mean June temperature and precipitation distributions associated with tundra vegetation and area burned are shown as histograms and box plots. For both temperature and precipitation, distributions of vegetation and area burned differ significantly based on a Kolmogorov-Smirnov test with N_fires_ = 232 degrees of freedom (p<0.01). Most fires occurred in areas with a mean June temperature of 6–10°C and a mean June precipitation of 20–30 mm. The general bias towards warm and/or dry portions of the total climate space suggests an overriding importance of low effective moisture for facilitating tundra burning.

High fire frequencies in the ancient shrub tundra prompt questions about the relative roles of vegetation (fuels) and climate (summer temperature and precipitation) in controlling fire regimes in the Shrub Tundra Zone and the implications of this natural experiment for understanding future Arctic environmental change. Climate is perhaps most often invoked to explain past changes in fire regimes. However, the influence of climate on the fire regime in the Shrub Tundra Zone is not straightforward. Near the end of *Betula* shrub dominance and afterwards (ca 11.5-9.0 ka BP), summer temperatures in northern Alaska may have approached or exceeded modern levels [20]. However, such a temperature rise cannot explain the increase in fire frequencies at the beginning of the Shrub Tundra Zone, ca 14.0-12.0 ka BP. In contrast, paleoclimate proxies [13] suggest that this period was characterized by cooler-than-present summers. Furthermore, lowered lake levels in interior Alaska indicate that effective moisture was lower than present throughout the Shrub Tundra Zone [21]. Because summer temperatures were cooler than modern, low effective moisture must have been a key factor facilitating the fuel drying necessary to maintain high fire activity within the ancient shrub tundra. The importance of low effective moisture for facilitating tundra burning is evident in the pattern of 232 tundra fires that burned in Alaska between CE 1950–2005. These fires were significantly skewed to tundra regions with relatively dry and/or warm summer climate conditions, i.e. with mean June precipitation between 20–30 mm and mean June temperature between 6–10°C (Fig. 4).

Given our current understanding of late glaciation and the early Holocene, increased burning in the Shrub Tundra Zone was not a simple function of climate change. The distinct increase in CHARs and CHAR peaks at the onset of the Shrub Tundra Zone suggests that vegetation was a key element facilitating fires. The tall growth form, small stem diameters, and highly resinous twigs of *B. glandulosa* [22] make it susceptible to fire on modern landscapes [23], and a widespread cover of *B. glandulosa* in the past would have created the continuity of flammable fuels necessary for fire spread. In addition, vigorous sprouting following fires [23] would have provided the regeneration necessary to sustain fire frequencies similar to those of modern boreal forests (Fig. 3). Based on paleo and modern relationships between tundra fire occurrence and corresponding climatic conditions, the role of fuels is central to understanding past and future shifts in tundra fire regimes. In the case of the Shrub Tundra Zone, the combination of abundant flammable fuels and low effective moisture overwhelmed the mitigating effects of low temperatures on landscape flammability.

Overall, paleorecords from northcentral Alaska imply that ongoing shrub expansion and climate warming will result in greater burning within northern tundra ecosystems. The geographic extent of fire-regime changes could be quite large, as shrubs are expected to expand over the next century in both herb and low shrub tundra ecosystems, which comprise 67% of circumpolar Arctic tundra [10], [15] (Fig. 1). Over this same period, annual temperatures in the Arctic are projected to increase between 3–5°C over land, lengthening the growing season and likely decreasing effective moisture (in spite of increased summer precipitation) [8]. How long might it take for the current shrub expansion to trigger a significant change in fire frequencies? Within the chronological limitations of our records, past shrub expansion and fire-regime changes at each site occurred within a few centuries (Fig. 2). The duration of this shift is consistent with the estimated rate of shrub expansion within a large area of northern Alaska [0.4% yr^−1^ for ca 200,000 km^2^; 10]. Based on a simple logistic growth model and the assumption of a constant expansion rate, Tape *et al.* [10] hypothesize that the ongoing shrub expansion in this region started roughly 125 years ago and should reach 100% of the region in another 125 years. Thus, if fuels and low effective moisture are major limiting factors for tundra fires, we predict that fire frequencies will increase across modern tundra over the next several centuries.

Although our fire-history records provide unique insights into the potential response of modern tundra ecosystems to climate and vegetation change, they are imperfect analogs for future fire regimes. First, ongoing vegetation changes differ from those of the late-glacial period: several shrub taxa (*Salix*, *Alnus*, and *Betula*) are currently expanding into tundra [10], whereas *Betula* was the primary constituent of the ancient shrub tundra. The lower flammability of *Alnus* and *Salix* compared to *Betula* could make future shrub tundra less flammable than the ancient shrub tundra. Second, mechanisms of past and future climate change also differ. In the late-glacial and early-Holocene periods, Alaskan climate was responding to shrinking continental ice volumes, sea-level changes, and amplified seasonality arising from changes in the seasonal cycle of insolation [13]; in the future, increased concentrations of atmospheric greenhouse gases are projected to cause year-round warming in the Arctic, but with a greater increase in winter months [8]. Finally, we know little about the potential effects of a variety of biological and physical processes on climate-vegetation-fire interactions. For example, permafrost melting as a result of future warming [8] and/or increased burning [24] could further facilitate fires by promoting shrub expansion [10], or inhibit fires by increasing soil moisture [24].

Despite these uncertainties, Alaskan paleorecords provide clear precedence of shrub-dominated tundra sustaining higher fire frequencies than observed in present-day tundra. The future expansion of tundra shrubs [10], [16] coupled with decreased effective moisture [8] could thus enhance circumpolar Arctic burning and initiate feedbacks that are potentially important to the climate system. Feedbacks between increased tundra burning and climate are inherently complex [3]–[5], but studies of modern tundra fires suggest the possibility for both short- and long-term impacts from (1) increased summer soil temperatures and moisture levels from altered surface albedo and roughness [24], and (2) the release soil carbon through increased permafrost thaw depths and the consumption of the organic layer [24], [25]. Given the importance of land-atmosphere feedbacks in the Arctic [26]–[28], the precedence of a fire-prone tundra biome should motivate further research into the controls of tundra fire regimes and links between tundra burning and the climate system.

## Materials and Methods

### Lake sediment cores

We reconstructed fire and vegetation history from macroscopic charcoal and palynological data preserved in sediments from four lakes in the southcentral Brooks Range (Fig. 1B). Ruppert Lake (3 ha; N 67°04′16″, W 154°14′45″; 230 m asl) and Xindi Lake (7 ha; N 67°04′42″, W 152°29′30″; 240 m asl) have records spanning late glaciation and the early Holocene (15-9 ka BP). Both sites are surrounded today by *Picea mariana* (black spruce) dominated boreal forest. Additionally, late-Holocene (last 5.5 ka BP) charcoal records from Ruppert, Code (2 ha; N 67°09′29″, W 151°51′40″; 250 m asl), and Wild Tussock (15 ha; N 67°07′40″, W 151°22′55″; 290 m asl) lakes provide information about fire regimes from the modern boreal forest [as defined by 17] for comparison with late-glacial and early-Holocene records.

Two parallel, overlapping sediment cores were collected from the center of each lake in summer 2001 (Code,), 2002 (Ruppert), and 2003 (Xindi, Wild Tussock) using a modified Livingstone-type piston corer [29] and sliced at 0.25–0.5 cm intervals in the laboratory. Subsamples of 1 cm^3^ were prepared at varying intervals for pollen analysis according to PALE protocols [30], and pollen was counted to a terrestrial sum >300 grains at 400–1000× magnification. For charcoal analysis, 3–5 cm^3^ subsamples were taken from contiguous core slices, soaked in sodium metaphosphate for 72 hours, washed through a 150 µm sieve, and bleached with 8% H_2_O_2_ for 8 hours. Charcoal was identified at 10–40× magnification based on color, morphology, and texture [31].

### Chronologies

Chronologies are based on accelerator mass spectrometry (AMS) ^14^C dates of *Betula* macrofossils, concentrated *Picea* pollen grains, and/or concentrated charcoal particles, and all ages are expressed as calibrated ^14^C years before present (CE 1950; Table S1). AMS ^14^C ages were calibrated using CALIB 5.0 and the IntCal 04 dataset [32]. Calibrated dates and corresponding confidence intervals represent the 50^th^, 2.5^th^ and 97.5^th^ percentiles of the cumulative probability density function of calibrated ages, respectively [33]. Chronologies were developed using a weighted cubic smoothing spline with the smoothing parameter determined by the average distance (cm) between dates, such that greater sampling resulted in a more flexible spline. The inverse of the 95% confidence interval of the calibrated ^14^C date was used for weighting.

Given the density of radiocarbon dates in and around the Shrub Tundra Zone, and that CHARs are sensitive to sedimentation rates, we evaluated whether general features of the CHAR series at Xindi and Ruppert lakes varied significantly when using 5–7 alternative age-depth models. In no case did high CHARs or the distinct peaks of the Shrub Tundra Zone disappear. Charcoal concentrations (pieces cm^−3^) are also high in this period, giving us confidence that the high CHARs reflect increased charcoal accumulation and are not chronological artifacts.

### Statistical treatment of charcoal data

Peaks in the charcoal accumulation rate (pieces cm^−2^ yr^−1^; CHAR) in lake sediment records have been shown both empirically [34] and through mechanistic models [35] to be associated with the local (0.5–1.0 km) occurrence of individual or multiple high-severity fires (“fire events”). Local fires introduce charcoal to a lake via airborne fallout and create distinct CHAR peaks that exceed variability around a low-frequency trend. This characteristic can be taken advantage of to infer when local fires occurred in the past. We estimated the timing of fire events in our charcoal records by removing low-frequency trends (i.e. “background”; reflecting changes in the rates of charcoal production, secondary transport, sediment mixing, and sediment sampling [31]) and applying a locally-defined threshold value that separates fire-related CHAR peaks (i.e. signal) from non-fire-related variability in CHARs (i.e. noise). Our approach accounts for changes in both the mean and variability of CHARs through time and the statistical nature of charcoal counts.

Prior to quantitative analysis, charcoal data were interpolated to constant 15-yr time steps, approximating the median temporal resolution of each record. Low-frequency trends in CHARs, *C_background_*, were modeled with a 500-yr running median, smoothed with a locally-weighted regression (also with a 500-yr window). We subtracted *C_background_* from the interpolated charcoal series to obtain a residual “peak” series, *C_peak_*. For each sample in each record, we identified charcoal peaks when *C_peak_* exceeded a sample-specific threshold value. Our threshold criterion assumes that fires create charcoal peaks that exceed *C_peak_* variations related to sediment mixing, sediment sampling, and analytical noise, and that this variability changes on time scales ≥500 years. Thus, for each 500-yr period, we assume that the distribution of *C_peak_* values contains two sub-populations: *C_noise_* and *C_fire_*. *C_noise_* is a normally-distributed population centered near 0 (i.e. *C_background_*); *C_fire_* samples are high CHARs exceeding variations in *C_noise_*, presumably caused by local fires. We used a Gaussian mixture model to identify the mean and variance of the *C_noise_* distribution [36], and we used the 99^th^ percentile of this distribution as the threshold value separating *C_fire_* from *C_noise_*. For all records, this procedure was done for each overlapping 500-yr period, producing a unique threshold for each sample. Individual thresholds for each sample were smoothed with a locally-weighted regression (to 500 yr). Finally, all peaks exceeding the locally-defined threshold were screened based on the original charcoal counts contributing to each peak. If the maximum count in a CHAR peak had a >5% chance of coming from the same Poisson-distributed population as the minimum charcoal count within the proceeding 75 years, then the “peak” was not identified [e.g. Charster user's guide, accessed September 2007, http://geography.uoregon.edu/gavin/charster/Analysis.html; 37]. Our methods are contained within the program *CharAnalysis*, written by PEH and freely available at http://CharAnalysis.googlepages.com.

### Quantifying fire regimes

We used dates of estimated fire events to calculate fire return intervals (years between fire events; FRIs), and we fit a two-parameter Weibull model to the distribution of FRIs within each vegetation zone using maximum likelihood techniques [38]. Each Weibull model passed a Kolmogorov-Smirnov goodness-of-fit test (p >0.10), and we estimated 95% confidence intervals for the Weibull scale, *b*, and shape, *c*, parameters based on 1000 bootstrapped samples from each population. Confidence intervals for the mean FRI were calculated in the same manner. We used a likelihood ratio test, based on likelihood values of the Weibull models, to test the null hypothesis that any two FRI distributions were similar [38], [39]. The probability of Type I Error, p, was estimated using a permutation test, and the null hypothesis was rejected if p<0.05.

### Climate space of modern tundra and tundra fires

The climate space occupied by modern tundra vegetation and tundra fires was quantified using tundra classification data from the circumpolar Arctic vegetation map [15], temperature and precipitation data from the Global Historical Climatology Network [W. Cramer. 2006 University of California-Berkeley, Integrative Biology and U.S. Geological Survey, Alaska Geographic Science Office. Accessed on-line in January 2007: http://agdc.usgs.gov/data/projects/hlct/hlct.html#A], and area burned data from the Alaska Fire Service [accessed on-line in January 2007: http://agdc.usgs.gov/data/blm/fire/index.html]. Climate data represent averages across variable periods, starting from 1888–1968 and generally ending in 1990. Each dataset was imported into a raster-based geographic information system with a 1 km^2^ cell size. Climate space was determined based on the average June precipitation and average June temperature values from all cells with: (1) CAVM classification of tundra, and (2) burned cells with a CAVM classification of tundra.

## Supporting Information

Table S1 Radiocarbon dates and calibrated ages for Ruppert, Xindi, Code, and Wild Tussock lakes.
(0.01 MB PDF)

Figure S1 High-frequency trends in the charcoal accumulation rate (CHAR) within the Boreal Forest Zone (5.5 ka BP - present) at Ruppert, Code, and Wild Tussock lakes. Red lines represent modeled variations in *C_noise_*, and plus marks identify peaks interpreted as local fire events, as in Fig. 2. Inferred fires from these sites were used to derive the boreal forest Weibull models presented in Fig. 3. See Materials and Methods for details.
(1.67 MB TIF)
