# Supplementary material for: Frequent Fires in Ancient Shrub Tundra: Implications of Paleorecords for Arctic Environmental Change
Source: PLoS One. 2008 Mar 5;3(3):e1744. doi: 10.1371/journal.pone.0001744 (PMC2254503; doi:10.1371/journal.pone.0001744)
Supplement: Table S1 — Radiocarbon dates and calibrated ages for Ruppert, Xindi, Code, and Wild Tussock lakes. (0.01 MB PDF) [file pone.0001744.s001.pdf]

Sample depth (cm)	Material Dated	Laboratory ID[1]	^{14}C date[2] (yr BP)	Calibrated date[3]	95% CI
Ruppert Lake					
16.60 - 17.60	concentrated charcoal	CAMS 106161	600 ± 100	594	475 - 743
26.48 - 27.20	concentrated charcoal	CAMS 110400	1170 ± 35	1088	1002 - 1190
30.5 - 31.5	concentrated charcoal	CAMS 106160	1150 ± 60	1065	904 - 1175
41.17 - 42.13	concentrated charcoal	CAMS 111400	1505 ± 40	1388	1266 - 1461
45.98 - 46.71	concentrated charcoal	CAMS 110401	1740 ± 35	1648	1542 - 1739
57.78 - 58.75	con. charcoal & *Picea* needle	CAMS 111401	2185 ± 40	2210	2104 - 2352
78.5 - 79.5	concentrated charcoal	CAMS 110948	3000 ± 60	3185	3029 - 3379
86.5 - 87.0	concentrated charcoal	CAMS 111402	3145 ± 35	3369	3285 - 3466
99.0 - 100.0	concentrated *Picea* pollen	CAMS 100063	3860 ± 45	4281	4155 - 4429
100.0 - 101.0	con. charcoal & *Picea* needle	CAMS 110949	3770 ± 40	4137	4004 - 4275
115.2 - 115.6	concentrated charcoal	CAMS 110402	5050 ± 45	5812	5720 - 5952
160.5 - 161.0	concentrated charcoal	CAMS 110950	6350 ± 110	7266	7077 - 7556
206.5 - 207.5	concentrated charcoal	CAMS 113762	7460 ± 110	8256	8082 - 8478
298.0 - 300.5	con. charcoal & *Betula* leafs	CAMS 122361	8710 ± 40	9654	9446 - 9750
324.5 - 326.5	concentrated charcoal	CAMS 111403	10220 ± 160	11939	11159 - 12549
380.5 - 381.5	concentrated charcoal	CAMS 110951	10740 ± 80	12820	12610 - 13239
423.9 - 427.4	concentrated charcoal	CAMS 122362	10870 ± 80	12860	12749 - 12952
Xindi Lake					
10.5 - 12.0	concentrated charcoal	CAMS 113558	1240 ± 70	1159	1036 - 1323
24.0 - 25.5	concentrated charcoal	CAMS 116226	3490 ± 35	3956	3940 - 3963
31.0 - 32.0	concentrated *Picea* pollen	CAMS 105876	4930 ± 90	5679	5472 - 5877
32.0 - 33.0	concentrated charcoal	CAMS 112145	4560 ± 120	5208	4860 - 5527
43.0 - 43.5	concentrated charcoal	CAMS 113559	4760 ± 70	5493	5377 - 5656
51.0 - 52.0	concentrated charcoal	CAMS 116227	5960 ± 60	7153	7144 - 7156
85.5 - 87.5	wood macrofossil	CAMS 106159	9585 ± 40	10907	10685 - 11083
127.0 - 127.5	concentrated charcoal	CAMS 114331	10180 ± 120	11844	11332 - 12330
167.5 - 168.5	concentrated charcoal	CAMS 114332	11800 ± 120	13648	13391 - 13903
183.5 - 184.5	concentrated charcoal	CAMS 114333	11570 ± 300	13456	12833 - 13961
Code Lake					
8.50 - 9.00	concentrated charcoal	CAMS 116841	405 ± 40	534	513 - 537
31.00 - 31.50	concentrated charcoal	CAMS 114723	1295 ± 35	1235	1182 - 1325
49.00 - 49.50	concentrated charcoal	CAMS 114724	2275 ± 30	2305	2266 - 2443
59.25 - 60.00	concentrated charcoal	CAMS 116840	2805 ± 40	3154	3104 - 3167
86.25 - 87.00	wood macrofossil	CAMS 80792	4155 ± 40	4691	4560 - 4833
96.50 - 97.50	concentrated charcoal	CAMS 116839	4875 ± 35	5742	5630 - 5746
123.00 - 123.50	wood macrofossil	CAMS 80794	6555 ± 40	7462	7367 - 7552
Wild Tussock Lake					
31.00 - 31.25	concentrated charcoal	CAMS 112143	1895 ± 45	1845	1737 - 1955
53.25 - 53.75	concentrated charcoal	CAMS 113763	2880 ± 60	3012	2820 - 3167
69.25 - 70.75	concentrated charcoal	CAMS 122363	3360 ± 35	3601	3516 - 3714
116.00 - 116.50	concentrated charcoal	CAMS 112144	4920 ± 70	5671	5461 - 5831
111.75 - 113.75	concentrated charcoal	CAMS 116228	4590 ± 50	5578	5572 - 5580
132.75 - 133.50	concentrated charcoal	CAMS 116229	5660 ± 120	6991	6833 - 7013

[1]CAMS: Center for Accelerator Mass Spectrometry, Lawrence Livermore National Laboratory, Livermore, CA.

[2]Conventional radiocarbon years before present (CE 1950) and standard deviation. [3]See Materials and Methods.
