# Supplementary figures and images for: Frequent Fires in Ancient Shrub Tundra: Implications of Paleorecords for Arctic Environmental Change
Source: PLoS One. 2008 Mar 5;3(3):e1744. doi: 10.1371/journal.pone.0001744 (PMC2254503; doi:10.1371/journal.pone.0001744)

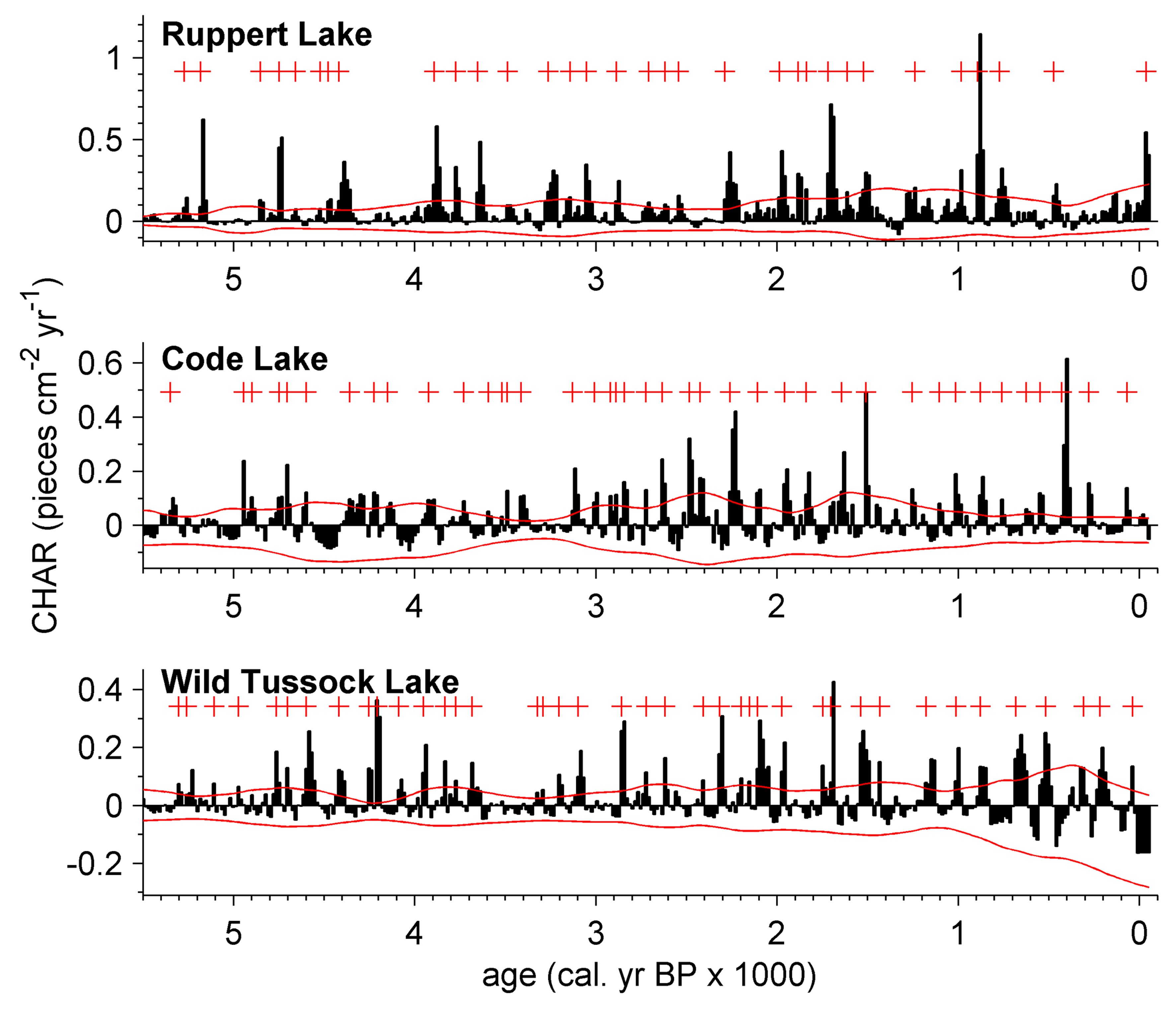

Supplement: Figure S1 — High-frequency trends in the charcoal accumulation rate (CHAR) within the Boreal Forest Zone (5.5 ka BP - present) at Ruppert, Code, and Wild Tussock lakes. Red lines represent modeled variations in Cnoise, and plus marks identify peaks interpreted as local fire events, as in Fig. 2. Inferred fires from these sites were used to derive the boreal forest Weibull models presented in Fig. 3. See Materials and Methods for details. (1.67 MB TIF) [file pone.0001744.s002.tif]
